# Storage stability of five steroids and in dried blood spots for newborn screening and retrospective diagnosis of congenital adrenal hyperplasia

**DOI:** 10.1371/journal.pone.0233724

**Published:** 2020-05-29

**Authors:** Nóra Grecsó, Anita Zádori, Ilona Szécsi, Ákos Baráth, Zsolt Galla, Csaba Bereczki, Péter Monostori

**Affiliations:** Metabolic and Newborn Screening Laboratory, Department of Pediatrics, University of Szeged, Szeged, Hungary; University of Pisa, ITALY

## Abstract

Congenital adrenal hyperplasia (CAH) is a severe inherited disorder of cortisol biosynthesis that is potentially lethal or can seriously affect quality of life. For the first time, we aimed to assess the stability of 21-deoxycortisol (21Deox), 11-deoxycortisol (11Deox), 4-androstenedione (4AD), 17-hydroxyprogesterone (17OHP) and cortisol (Cort), diagnostic for CAH, in dried blood spots (DBSs) during a 1 year storage at different temperatures. Spiked DBS samples were stored at room temperature, 4 °C, -20 °C or -70 °C, respectively and analyzed in triplicates using liquid chromatography–tandem mass spectrometry at Weeks 0, 1, 2, 3 and 4, Month 6 and Year 1. Analyte levels within ±15% *vs* the baseline were considered stable. Our observations show that 21Deox, 4AD and 17OHP were not significantly changed for 1 year even at room temperature at either analyte levels. In contrast, Cort required storage at 4 °C, -20 °C or -70 °C for long-term stability, being significantly decreased at room temperature from Month 6 (*p*<0.01) in both the 30(60) nM and the 90(180) nM samples. 11Deox was significantly decreased at room temperature at Year 1 (*p*<0.01) and only in the 30(60) nM samples. Thus, all biomarkers were stable for up to 1 year at 4 °C, -20 °C or -70 °C and at least for 4 weeks at room temperature. These findings have implications for analyses of stored DBS samples in 2^nd^-tier assays in newborn screening and for retrospective CAH studies.

## Introduction

Congenital adrenal hyperplasia (CAH; OMIM 201910, OMIM 202010) is a severe inherited disorder of cortisol biosynthesis [1,2]. The underlying enzyme defect can result in a disturbed sodium homeostasis with hyponatremia and hyperkalemia which can cause death in early infancy [1,2]. The accumulated steroid precursors are in part converted to androgens, which can additionally cause prenatal virilization in affected girls and postnatal androgen excess in both males and females, resulting in a markedly reduced quality of life [1,2].

In order to achieve a timely diagnosis of this possibly lethal disorder, dried blood spots (DBSs) [3] are used for the newborn screening (NBS) of CAH for the measurement of the 17-hydroxyprogesterone (17OHP) level by means of time-resolved, dissociation-enhanced, lanthanide fluorescence immunoassay (DELFIA^®^) [1,2]. Due to the relatively high number of false-positives and the resulting low positive predictive value in the 1^st^-tier testing, the confirmation of CAH with a second assay is necessary [1,2]. Second-tier testing using liquid chromatography–tandem mass spectrometry (LC-MS/MS) is preferable which uses the same DBS specimen as in the primary screening [1,2,4–6].

For this approach, it has to be assumed that steroid levels in the DBS specimen are sufficiently stable and are representative for the time of blood sampling [7–9]. However, current literature on the stability of the steroids in DBS is very limited, dealing exclusively with 17OHP [10–16] or cortisol (Cort) [17]. Data on the DBS stability of 21-deoxycortisol (21Deox) and 11-deoxycortisol (11Deox), which are specific biomarkers of the two main types of CAH, the 21-hydroxylase and 11β-hydroxylase deficiencies [1,2]; or that of 4-androstenedione (4AD) have not been published yet.

Long-term stability data on these biomarkers could additionally extend the current knowledge regarding the retrospective analysis of stored DBS samples. As an example, retrospective analysis of NBS specimens from symptomatically diagnosed patients in countries without NBS for CAH [18] could provide valuable information whether CAH would have already been detected at newborn age. Such results could then be used as evidence to facilitate local healthcare providers to include CAH in the NBS panel. Moreover, retrospective analysis of stored DBS may be an important tool in clarifying if a deceased child had been suffering from CAH or not. This may promote awareness and family planning in affected families, which is considered as an important complementary outcome of NBS, in addition to the timely diagnosis of affected children [19].

We therefore aimed to assess the time course of the potential alterations of five steroids, diagnostic for CAH, during a 1 year storage in DBS. To elucidate possible effects of the storage temperature, measurements were performed on specimens stored at room temperature, 4 °C, -20 °C or -70 °C, respectively.

## Materials and methods

### Reagents

Unlabelled Cort, 21Deox, 11Deox, 4AD and 17OHP were purchased from Sigma-Aldrich (St. Louis, MO, USA); deuterated internal standards (ISs) d_4_-Cort, d_8_-21Deox, d_2_-11Deox, d_5_-4AD and d_8_-17OHP from Cambridge Isotope Laboratories (Andover, MA, USA); acetonitrile (LC-MS grade) from Merck (Darmstadt, Germany); methanol (LC-MS grade) and formic acid (LC-MS grade) from Honeywell (Charlotte, NC, USA). Ultrapure water (18.2 MΩ.cm), filtered through a 0.22 μm pore size membrane, was obtained from a Merck Millipore Direct-Q 3 UV system (Billerica, MA, USA).

### Preparation of standard and IS stock, intermediate and working solutions

Unlabelled standards and deuterated ISs were dissolved in methanol separately to obtain 5 mM stock solutions. An intermediate solution mix of unlabelled standards was obtained by mixing stock solutions and diluting with methanol/water 50/50 (v/v) to give final concentrations of 5 μM each for 21Deox, 11Deox, 4AD and 17OHP and 10 μM for Cort (indicated as 5(10) μM). An intermediate solution mix of deuterated ISs was obtained by mixing IS stock solutions and diluting with methanol/water 50/50 (v/v) to give final concentrations of 100 nM for d_4_-Cort, 75 nM for d_8_-21Deox and 15 nM each for d_2_-11Deox, d_5_-4AD and d_8_-17OHP. Stock solutions and intermediate solution mixes were stored in aliquots at -70 °C. The IS working solution (composition: 10 nM d_4_-Cort, 7.5 nM d_8_-21Deox and 1.5 nM each of d_2_-11Deox, d_5_-4AD and d_8_-17OHP) was prepared fresh daily by a 10-fold dilution of the intermediate solution mix of deuterated ISs with acetonitrile/water 80/20 (v/v).

### Preparation of the DBS samples, duration and conditions of storage

Heparinized blood form a single healthy volunteer (author I.SZ.) was centrifuged at 1000 x g for 15 min, and the erythrocytes were washed three times with phosphate-buffered saline (serum and supernatant were discarded). The erythrocytes were mixed with commercially available steroid-depleted serum (BBI Solutions, Crumlin, UK) to produce steroid-depleted blood with a hematocrit of 50%. Thereafter, the steroid-depleted blood was subjected to three freeze-thaw cycles at -70 °C to ensure homogeneity.

For the preparation of the calibrators, an aliquot of the steroid-depleted blood was spiked with the intermediate solution mix of unlabeled standards to obtain a concentration of 250(500) nM for steroids (Cort). Calibrators were prepared via mixing appropriate volumes of the 250(500) nM sample or the respective previous calibrator gently but thoroughly with steroid-depleted blood. The 0(0) nM calibrator contained steroid-depleted blood without spiking. Final concentrations of the DBS calibrators were as follows: 0(0), 2(4), 5(10), 10(20), 25(50), 75(150) and 125(250) nM for steroids (Cort).

For the preparation of the QCs and test samples, aliquots of the steroid-depleted blood were spiked with equal volumes of spiking solutions in saline (corresponds to a 100-fold dilution of the analytes) and mixed gently but thoroughly. Final concentrations of the QCs were 15(30), 30(60) and 90(180) nM for steroids (Cort), respectively. The spiked test samples were prepared in levels 30(60) and 90(180) nM for steroids (Cort), respectively.

Thereafter, the samples were spotted onto Ahlstrom-Munksjö TFN filter paper cards (Ahlstrom-Munksjö Germany GmbH, Bärenstein, Germany) and dried at room temperature for 24 h. All DBS calibrators, QCs and test samples were stored in sealed aluminium bags with silica gel desiccants. The calibrators and the QCs were stored at -70 °C and were used for the quantification of all test samples, regardless of the storage temperature (room temperature, 4 °C, -20 °C or -70 °C), in line with the paper of Elvers et al [14]. For test samples stored frozen, repeated freeze-thaw cycles were avoided. Measurement time points (storage durations) were Weeks 0, 1, 2, 3 and 4, Month 6 and Year 1. All test samples were prepared as triplicates at each time point. Analytes remaining within ±15% *vs* the baseline were considered stable in line with recommendations [20].

All procedures followed were in accordance with the ethical standards of the responsible committee on human experimentation (institutional and national) and with the Helsinki Declaration of 1975, as revised in 2000. The study was approved by the Ethical Committee of the University of Szeged (139/2018-SZTE). The whole blood used for the preparation of the calibrators, QCs and spiked test samples originated from one of the authors (I.SZ.); therefore, no written consent was obtained. However, information on study characteristics and possible risks of blood sampling were verbally discussed among the authors beforehand and were documented in the internal protocol file.

### Sample preparation and instrumentation

The sample preparation procedures have been published previously [21]. Briefly, two spots 4.7 mm in diameter (corresponding to approx. 13.6 μl blood) of each of the DBS calibrators, QCs and test samples were extracted with the IS working solution for 45 min at ambient temperature in 96-well microtiter plates. Following centrifugation, the supernatant was transferred to a second microtiter plate and dried at 35 °C in a gentle flow of air for 45 min. The underivatized residues were reconstituted in 45 μl of a methanol/water mixture (20:80 v/v). The levels of Cort, 21Deox, 11Deox, 4AD and 17OHP were measured with a gradient LC-MS/MS method, which had been previously validated using External Quality Control specimens and clinical DBS samples with inter-day variabilities of approx. 10-15% [21]. The system consisted of a PerkinElmer Flexar UHPLC system (two FX-10 binary pumps, solvent manager with a degasser, autosampler and thermostatic oven; all PerkinElmer Inc., Waltham, MA, USA), combined with an AB SCIEX QTRAP 5500 MS/MS triple quadrupole mass spectrometer, and controlled by Analyst 1.6.1 software (both AB SCIEX, Framingham, MA, USA). Chromatographic separation was performed on a Phenomenex Kinetex XB-C18 100x3.0 mm, 2.6 μm core-shell analytical column and a SecurityGuard Ultra Cartridge guard column (both Phenomenex, Torrance, CA, USA). Eluent A consisted of ultrapure water (18.2 MΩ.cm, filtered through a 0.22 μm pore size membrane) plus 0.1% formic acid (LC-MS grade). Eluent B consisted of methanol plus 0.1% formic acid (both LC-MS grade). Results of the LC-MS/MS analysis are reported in nM.

### Statistical analysis

Statistical comparisons were performed by using repeated-measures two-way analysis of variance (ANOVA), followed by Dunnett’s multiple comparisons test, using GraphPad Prism software (GraphPad Software, La Jolla, CA, USA). Results are reported as means±standard deviation (SD). *p* values <0.01 were considered significant.

## Results

**Fig 1:**
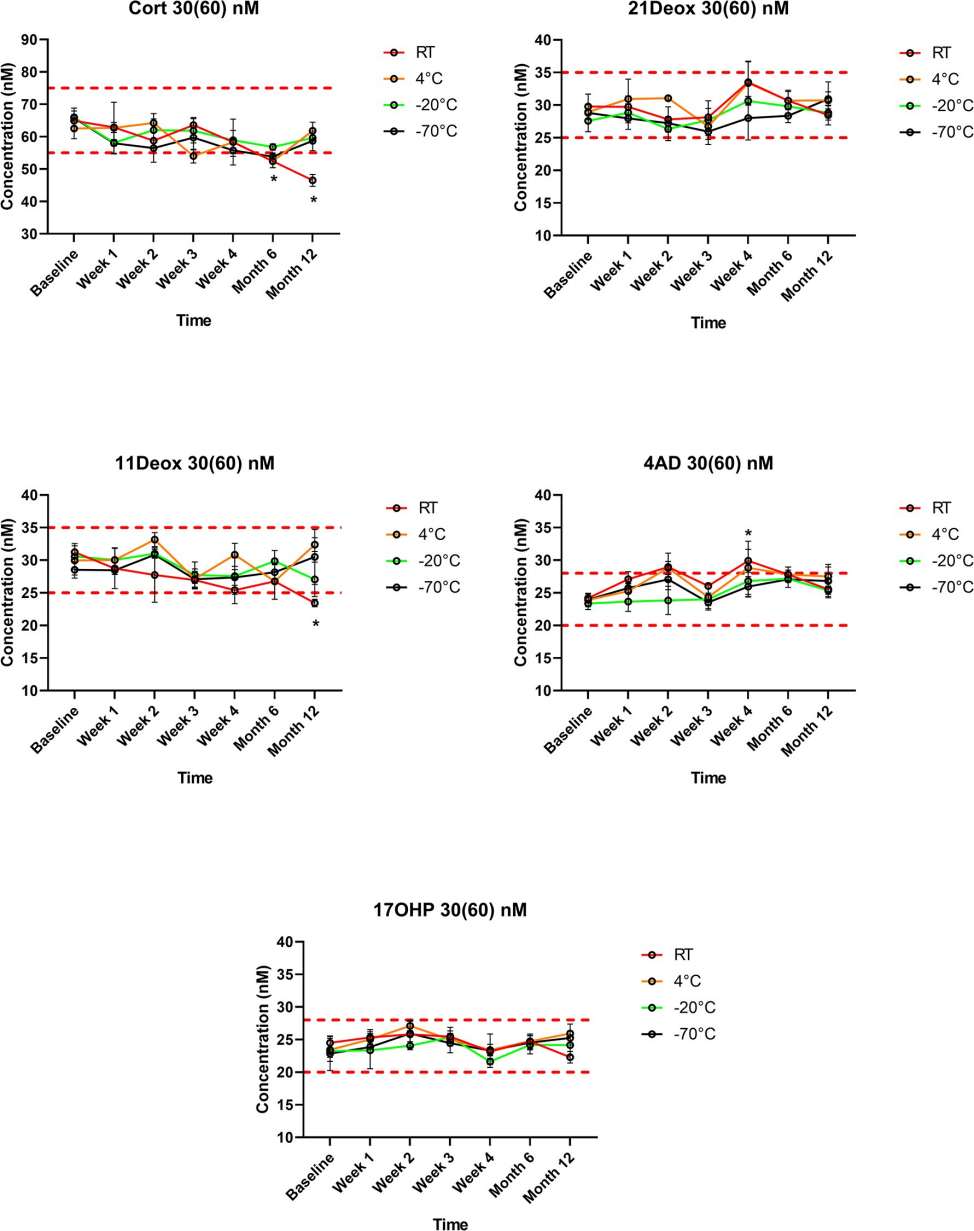
Steroid concentrations in the 30(60) nM test samples during storage within 1 year at different temperatures (*n* = 3 for each data point). Values are given as means±SD. Cort: cortisol; 21Deox: 21-deoxycortisol; 11Deox: 11-deoxycortisol; 4AD: 4-androstenedione; 17OHP: 17-hydroxyprogesterone; RT: room temperature. Dashed red lines indicate upper and lower cutoffs of the predetermined ranges (±15% *vs* Week 0), within which analytes were considered stable. * *p*<0.01 *vs* the baseline at room temperature. Error bars for SD values smaller than the respective symbol size are not visible.

**Fig 2:**
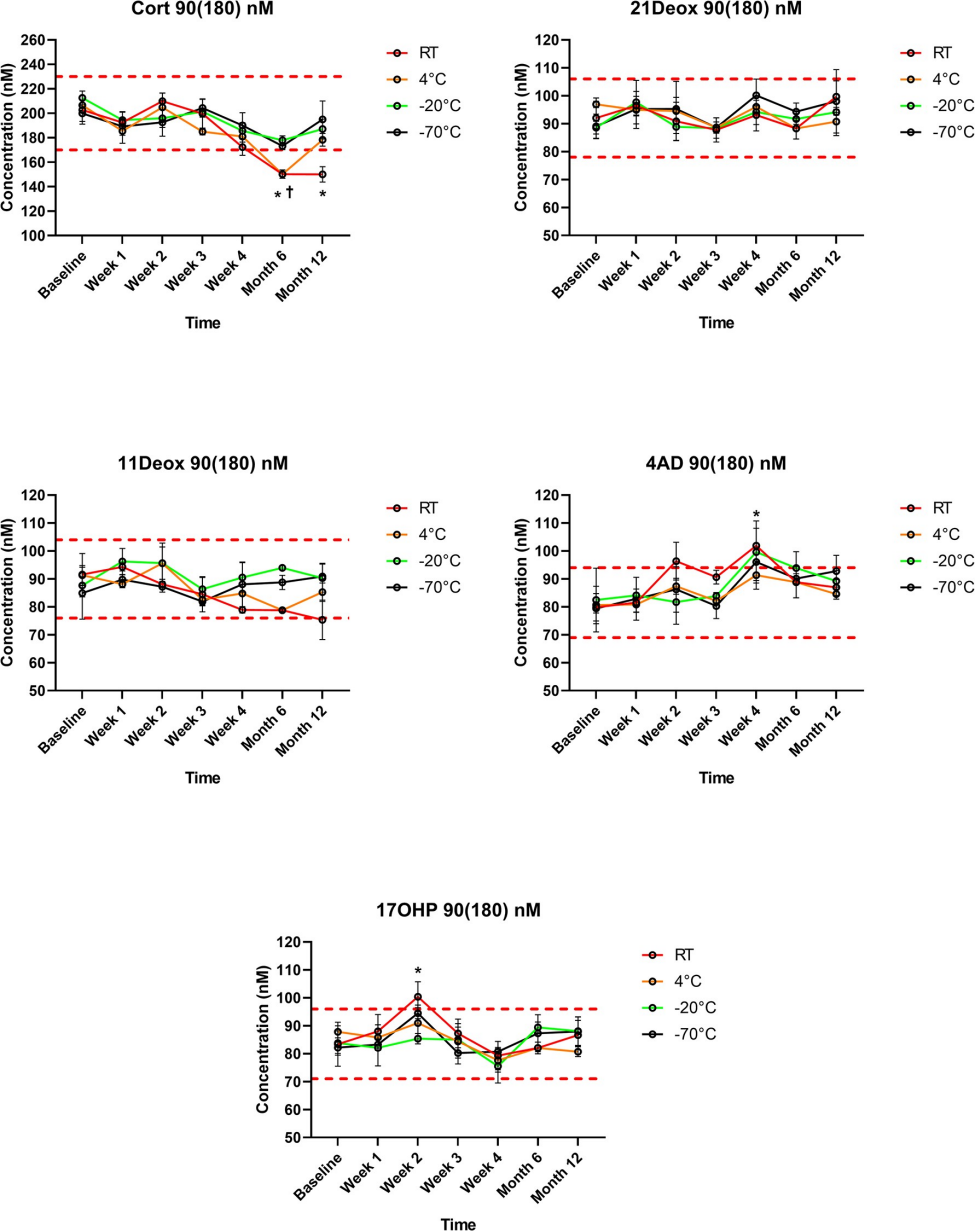
Steroid concentrations in the 90(180) nM test samples during storage within 1 year at different temperatures (*n* = 3 for each data point). Values are given means±SD. Cort: cortisol; 21Deox: 21-deoxycortisol; 11Deox: 11-deoxycortisol; 4AD: 4-androstenedione; 17OHP: 17-hydroxyprogesterone; RT: room temperature. Dashed red lines indicate upper and lower cutoffs of the predetermined ranges (±15% *vs* Week 0), within which analytes were considered stable. * *p*<0.01 *vs* the baseline at room temperature; † *p*<0.01 *vs* the baseline at 4 °C. Error bars for SD values smaller than the respective symbol size are not visible.

21Deox in DBS was stable during the study period, regardless of the storage temperature. Also 17OHP remained within the predetermined ±15% range, apart from significantly elevated values at a single measurement point (Week 2) at room temperature in the 90(180) nM samples (20% increase, *p*<0.01). When DBSs were stored at both -20 °C or -70 °C, Cort levels remained within the range throughout the 1 year interval, despite a slight but non-significant decreasing tendency in the 30(60) nM samples at both temperatures. However, in specimens stored at room temperature, Cort concentrations became significantly lower than the cutoff at Month 6 and at Year 1 in both the 30(60) nM and the 90(180) nM samples (28% and 26% decrease at Year 1, respectively; both *p*<0.01). At 4 °C, significantly lower Cort levels were measured only in the 90(180) nM samples at Month 6 (25% decrease, *p*<0.01), but not at Year 1. 11Deox was within the range for 1 year when stored at 4 °C, -20 °C or -70 °C, and was significantly decreased at Year 1 at room temperature in the 30(60) nM samples (18% decrease *p*<0.01). 4AD concentrations remained in the range, apart from significantly elevated values at a single measurement point (Week 4) at room temperature in both the 30(60) nM and the 90(180) nM samples (19% and 21% increase at Year 1, respectively; both *p*<0.01) (Figs 1 and 2, S1 Table). Using an external QC sample, variation during the 1-year study was found to be <9% for all analytes (S1 Table).

## Discussion

CAH is a severe and potentially life-threatening disorder where an early diagnosis is crucial for a timely initiation of treatment. The stability of Cort, 4AD and 17OHP levels have been previously assessed in various sample matrices including plasma, serum, saliva, hair or urine [22–26]. Storage durations in these studies ranged from 72h to 2 years; the storage temperature varied from -70 °C to ambient [22–26].

NBS for CAH is performed in several countries to facilitate early recognition, treatment and the prevention of serious morbidity and mortality [1,2]. However, the DBS, not plasma, serum, saliva, hair or urine, is the standard sample type in NBS [8,9]. A very limited number of papers on stability in DBS are available, evaluating 17OHP [10–16] or Cort [17]. There have been no reports on the stability of 21Deox, 11Deox or 4AD in DBS. According to the literature, 17OHP in DBS is stable for at least one year at room temperature [10–13] or for up to 2 years when stored frozen at -20 °C or -80 °C [14] or at 4 °C [15]. Using forced degradation at 37 °C, 17OHP was already decreased after 1 month [16]. The only paper on Cort in DBS reported that Cort from a single healthy volunteer remained unchanged after 1 month at room temperature [17]. Of note, the analytical techniques in these previous studies (radioimmunoassay, DELFIA, HPLC-diode array detection) are reportedly prone to cross-reactions and/or have relatively low selectivities [2,27] when compared with LC-MS/MS, which is the technique of choice for the determination of steroids in CAH [1,2,4–6].

The present study is the first to evaluate the stability of 21Deox, 11Deox or 4AD in DBS, together with Cort and 17OHP using a clinically validated LC-MS/MS method [21]. 21Deox was found to be stable throughout the 1 year study period, regardless of both the storage temperature and the concentration (Figs 1 and 2). 11Deox was also stable for 1 year at 4 °C, -20 °C or -70 °C; a significant decrease was only seen at room temperature at Year 1 and only in the 30(60) nM samples (*p*<0.01) (Figs 1 and 2). These new findings are important in the view that 21Deox (produced from the 11β-hydroxylation of 17OHP) and 11Deox are specific biomarkers of the two main subtypes of CAH, the 21-hydroxylase and 11β-hydroxylase deficiencies which account for >99% of all cases [1,2,4].

17OHP, the 1^st^-tier biomarker in CAH screening, was stable in DBS for 1 year (Figs 1 and 2), in line with previous studies [10–15]. 4AD concentrations were within the allowed range prior to and after Week 4 (Figs 1 and 2). Thus, elevated values of 4AD at Week 4 at both analyte levels, as well as higher 17OHP concentrations at Week 2 in the 90(180) nM samples seem to be only of statistical significance, rather than a sign of a real alteration with clinical relevance. Cort levels, when stored frozen at -20 °C or -70 °C, remained within the range in the study period despite an apparent slight decrease in the 30(60) nM samples. However, when stored at room temperature, Cort became significantly lower than the cutoff at Months 6 and 12 at both analyte levels. At 4 °C, lower Cort levels were only seen in the 90(180) nM samples and solely at Month 6, but not at Year 1, suggesting that this may be due to casual variation (Figs 1 and 2). Such random analytical alterations may reportedly be correctable by the application of ISs directly onto the filter paper. This approach has been described in previous papers for analytes other than steroids [28–30].

Further limitations of the present study include the relatively low number of replicates and the fact that more measurement time points would have allowed following the alterations more precisely. Even if an additional measurement had been planned for Month 3, a reliable acquisition was not possible due to an instrument breakdown and lengthy service process. In addition, stability testing of real patient samples should be considered in the future to elucidate other potential confounding factors such as steroid-binding proteins [31], despite the relatively large amount of blood necessary for prolonged storage experiments. Our results are suggested to be confirmed by the individual laboratories in their own settings, together with the assessment of potentially confounding factors, such as temperature and humidity [16].

One clinical implication of the present study is that the examined biomarkers of CAH are now proved to be stable in DBS for at least 4 weeks, allowing sufficient time to use NBS samples, generally stored at room temperature [9], in 2^nd^-tier assays and re-testing in the short-term. Second-tier testing is commonly performed within 7 days from blood sampling, whereas the salt-wasting phenotype of CAH normally presents within 2-3 weeks of age without timely diagnosis and treatment [32]. Although similar time frames arecommonly used in NBS for CAH, there have been no previous reports with experimental evidence on their appropriateness. For long-term storage (>4 weeks), suspected or confirmed patient samples are suggested to be stored frozen at -20 °C or -70 °C to allow for using them in method development, patient monitoring and quality assurance purposes.

Furthermore, our stability results extend the current knowledge regarding the retrospective analysis of stored DBS samples, including those from symptomatically diagnosed patients in countries without NBS for CAH [18]. Such results may facilitate local healthcare providers to include CAH in the NBS panel, as well as promote awareness and family planning in affected families [19]. However, these assumptions have to be confirmed via retrospective analysis of real patient samples.

## Conclusions

For the first time, storage stabilities of 21Deox, 11Deox, Cort, 4AD and 17OHP levels were simultaneously evaluated in DBS using a clinically validated LC-MS/MS method. Experimental evidence is provided that the assessed biomarkers are stable for at least 4 weeks even at room temperature, allowing sufficient time to use NBS samples in 2^nd^-tier assays and re-testing. As concerns long-term storage for up to 1 year, concentrations of 21Deox, 4AD and 17OHP were found to be stable. Cort and 11Deox levels remained within the ±15% range when stored at 4 °C, -20 °C or -70 °C. Cort was decreased at Months 6 and 12 when stored at room temperature. 11Deox was decreased at Month 12 at room temperature only in the 30(60) nM samples. These new findings have implications for analyses of stored DBS samples in 2^nd^-tier assays in NBS and for various retrospective studies on CAH.

## Supporting information

S1 Text Preparation of stock solutions and dried blood spot (DBS) calibrators, Quality Controls (QCs) and test samples. (DOCX)

S1 Table Stability of the external QC sample throughout the study. (XLSX)

## Abbreviations

11Deox: 11-deoxycortisol
17OHP: 17-hydroxyprogesterone
21Deox: 21-deoxycortisol
4AD: 4-androstenedione
CAH: congenital adrenal hyperplasia
Cort: cortisol
DBS: dried blood spot
IS: internal standard
LC-MS/MS: liquid chromatography-tandem mass spectrometry
NBS: newborn screening
QC: quality control
