# Supplementary material for: Storage stability of five steroids and in dried blood spots for newborn screening and retrospective diagnosis of congenital adrenal hyperplasia
Source: PLoS One. 2020 May 29;15(5):e0233724. doi: 10.1371/journal.pone.0233724 (PMC7259505; doi:10.1371/journal.pone.0233724)
Supplement: S1 Text — (DOCX) [file pone.0233724.s001.docx]

**S1  Text**

**Manuscript title**

**Storage stability of five steroids and corresponding analyte ratios in dried blood spots for newborn screening and retrospective diagnosis of congenital adrenal hyperplasia**

Nóra Grecsó^1*^, Anita Zádori^1^, Ilona Szécsi^1^, Csaba Bereczki^1^, Péter Monostori^1^

^1^ Metabolic and Newborn Screening Laboratory, Department of Pediatrics, University of Szeged, Szeged, Korányi fasor 14‑15, H‑6720, Hungary

*** Corresponding author**

E‑mail: grecso.nora.ildiko@med.u-szeged.hu (NG)

**S1 Text: Preparation of stock solutions and dried blood spot (DBS) calibrators, Quality Controls (QCs) and test samples**

*Preparation of standard and internal standard stock, intermediate and working solutions*

Unlabelled standards (cortisol (Cort), 21‑deoxycortisol (21Deox), 11‑deoxycortisol (11Deox), 4‑androstenedione (4AD) and 17‑hydroxyprogesterone (17OHP)) and deuterated internal standards (ISs; d_4_‑Cort, d_8_‑21Deox, d_2_‑11Deox, d_5_‑4AD and d_8_‑17OHP) were dissolved in methanol separately to obtain 5 mM stock solutions. An intermediate solution mix of unlabelled standards was obtained by mixing stock solutions and diluting with methanol/water 50/50 (v/v) to give final concentrations of 5 μM each for 21Deox, 11Deox, 4AD and 17OHP and 10 μM for Cort (indicated as 5(10) μM). An intermediate solution mix of deuterated ISs was obtained by mixing IS stock solutions and diluting with methanol/water 50/50 (v/v) to give final concentrations of 100 nM for d_4_‑Cort, 75 nM for d_8_‑21Deox and 15 nM each for d_2_‑11Deox, d_5_‑4AD and d_8_‑17OHP. Stock solutions and intermediate solution mixes were stored at ‑70 °C. The IS working solution (composition: 10 nM d_4_‑Cort, 7.5 nM d_8_‑21Deox and 1.5 nM each of d_2_‑11Deox, d_5_‑4AD and d_8_‑17OHP) was prepared fresh daily by a 10‑fold dilution of the intermediate solution mix of deuterated ISs with acetonitrile/water 80/20 (v/v).

*Preparation of DBS calibrators, QCs and test samples*

Heparinized blood was centrifuged at 1000 x g for 15 min, and the erythrocytes were washed three times with phosphate-buffered saline (the serum and supernatant were discarded). The erythrocytes were mixed with commercially available steroid-depleted serum (BBI Solutions, Crumlin, UK) to produce steroid-depleted blood with a hematocrit of 50%. Thereafter, the steroid-depleted blood was subjected to three freeze-thaw cycles at ‑70 °C to ensure homogeneity.

For the preparation of the calibrators, an aliquot of the steroid-depleted blood was spiked with the intermediate solution mix of unlabeled standards to obtain a concentration of 250(500) nM for steroids (Cort). Calibrators were prepared via mixing appropriate volumes of the 250(500) nM sample or the respective previous calibrator gently but thoroughly with steroid-depleted blood. The 0(0) nM calibrator contained steroid-depleted blood without spiking. Final concentrations of the DBS calibrators were 0(0), 2(4), 5(10), 10(20), 25(50), 75(150) and 125(250) nM for steroids (Cort). For the preparation of the QCs and test samples, aliquots of the steroid-depleted blood were spiked with equal volumes of spiking solutions in saline (corresponds to a 100‑fold dilution of the analytes) and mixed gently but thoroughly. Final concentrations of the QCs were 15(30), 30(60) and 90(180) nM for steroids (Cort), respectively. The test samples were prepared in levels 30(60) and 90(180) nM for steroids (Cort), respectively.

Thereafter, the samples were spotted onto Ahlstrom 226 filter paper cards (Ahlstrom-Munksjö Germany GmbH, Bärenstein, Germany) and dried at room temperature for 24 h. All DBS calibrators, QCs and test samples were stored in sealed aluminium bags with desiccants. The calibrators and the QCs were stored at ‑70 °C. The test samples were stored at room temperature, 4 °C, ‑20 °C or ‑70 °C, respectively.
